# Malaria reporting timeliness analysis and factors associated with delayed notification, 2018–2022, Nepal

**DOI:** 10.1371/journal.pgph.0003589

**Published:** 2024-08-26

**Authors:** Shashi Kandel, Gokarna Dahal, Rudra Prasad Marasini, Krishna Prasad Paudel, Ashna Parajuli, Susmita Thapa, Rizu Aryal, Kanlaya Jongcherdchootrakul, Phanthanee Thitichai

**Affiliations:** 1 Department of Health Services, Epidemiology and Disease Control Division, Ministry of Health and Population, Kathmandu, Nepal; 2 Ministry of Health and Population, Kathmandu, Nepal; 3 Department of Military and Community Medicine, Phramongkutklao College of Medicine, Bangkok, Thailand; 4 Department of Disease Control, Division of Epidemiology, Field Epidemiology Training Program, Ministry of Public Health, Nonthaburi, Thailand; Straive, PHILIPPINES

## Abstract

In order to monitor public health trends and identify disease outbreaks early, efficient and reliable notification and surveillance systems are essential. Nepal uses a 1-3-7 malaria surveillance approach. The Short Message System (SMS) -based system for timely notification has been established. However, knowledge gaps exist regarding the timeliness of notification, treatment initiation, and case-based investigations. Hence, this study identifies the timeliness of notification and factors associated with delayed notification. This study used a cross-sectional approach and used secondary malaria surveillance data from Nepal’s national malaria elimination program for the period of 2018 to 2022. The study revealed that the majority (79.9%) of malaria cases were male, with a male-to-female ratio of 3.96:1. Occupation was found to be significantly associated with delayed notification. Repatriate workers had 0.60 times lower odds of experiencing delayed notification compared to the reference occupation. Similarly, individuals diagnosed in the Sudurpaschim and Lumbini provinces had significantly lower odds (0.48 and 0.38, respectively) of encountering delayed notification compared to the reference province. Furthermore, relying on a single laboratory tool for malaria diagnosis (either RDT or microscopy only) was significantly associated with delayed notification. Individuals diagnosed solely with RDT or microscopy had 2.04 and 1.79 times higher odds of experiencing delayed notification, respectively, compared to those diagnosed using both laboratory tools. This study provides insight into the timeliness of surveillance system approach by assessing delayed notification and the factors associated with it. No delays are identified in median notification, treatment time and in case investigation. Improvement in the timeliness of malaria reporting over the years was observed. Provinces with high burden of malaria and repatriate workers showed lower delayed notification and conversely, cases diagnosed with single laboratory tool showed delayed notification time.

## Introduction

Malaria is a life-threatening disease caused by protozoan parasite of the genus Plasmodium and transmitted through the bite of infected female Anopheles mosquitoes to humans [1]. In 2021, it was estimated that there were approximately 247 million cases of malaria and 619,000 related deaths globally. The WHO South-East Asia region had nine countries with endemic malaria, accounting for 5.4 million cases, which represents 2% of the global burden of malaria cases. The Southeast Asia Region is the region with the second-highest estimated malaria burden globally. The number of cases has been significantly reduced in this region, falling from 22.8 million in 2000 to 5.4 million in 2021, a 76% decrease. About 40% of all cases in the region were due to plasmodium vivax [2].

Malaria was a significant health issue in lowland Nepal until the latter half of the 20th century. Surveys conducted in 1925 in the inner Terai region revealed high spleen rates of 80% in children. It was estimated that there were 2 million malaria cases annually, with a fatality rate of 10–15% among these cases [3]. Since 1985, Nepal has experienced a steady decline in malaria burden. Factors contributing to this improvement include overall advancements in social determinants of health, the widespread use of combo-rapid diagnostic tests (RDTs), availability of effective antimalarial drugs like chloroquine, primaquine, and artemisinin-based combination therapy (ACT) in public healthcare facilities, and high coverage of long-lasting insecticidal nets (LLINs) in targeted areas [3, 4]. Over the years, Nepal witnessed fluctuations in imported and indigenous malaria cases. Indigenous cases showed a general decrease over the years, indicating progress in controlling local transmission [5]. Though the total number of cases are declining, the net number of imported cases grew by roughly 40 to 58% between the year 2009 and 2018 [3]. In order to monitor public health trends and identify disease outbreaks early, efficient and reliable notification and surveillance systems are essential [6].

**Fig 1:**
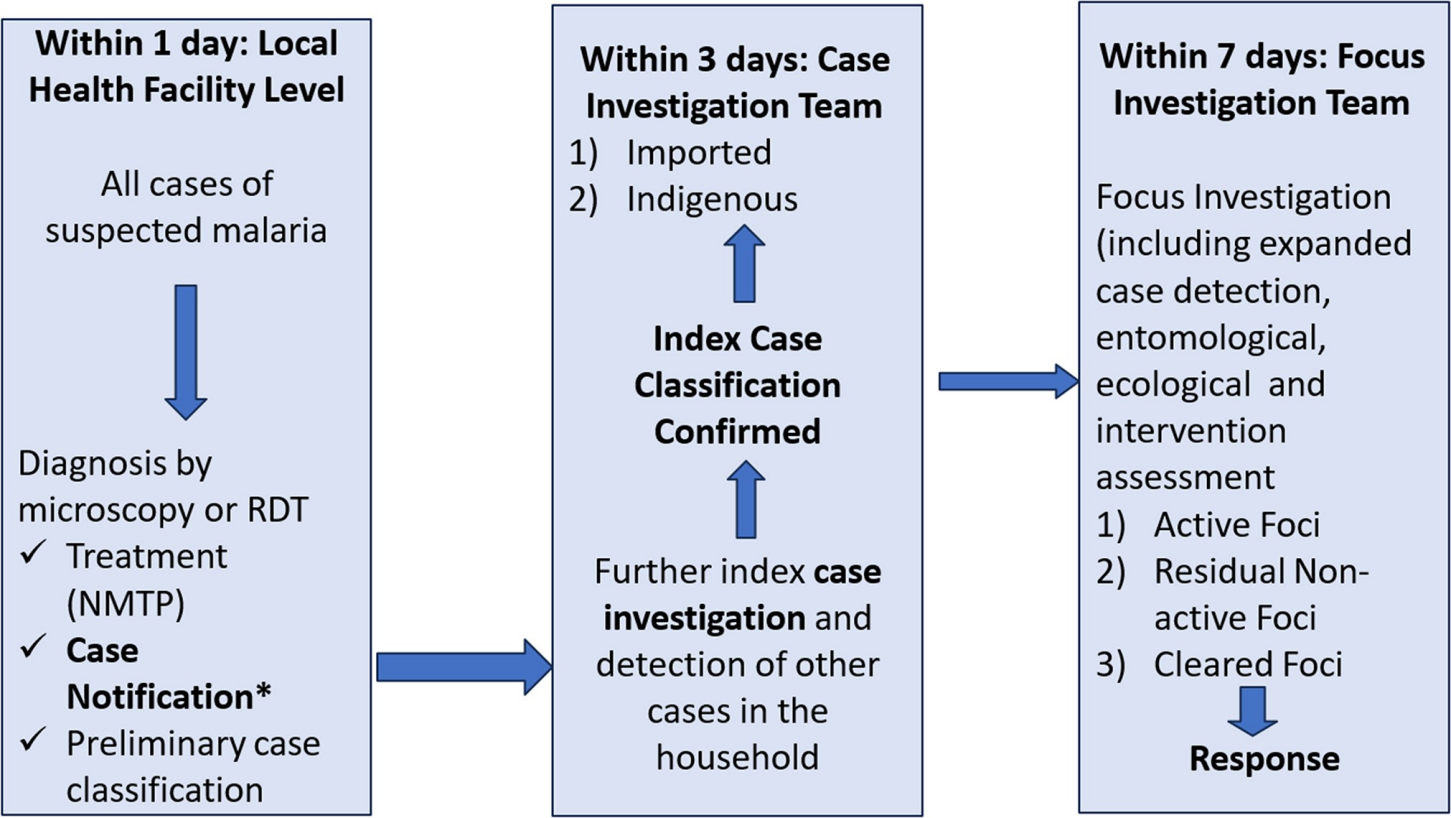
1-3-7 approach to malaria surveillance. Source: National Malaria Surveillance Guidelines 2019, Nepal.

Nepal has established a malaria surveillance system that aims to receive individual case notifications within 24 hours of detection from various sources, including public, private, and community sectors via a free Short Message Service (SMS) based system. The timely notification of cases is crucial for triggering prompt case investigations, which are expected to be initiated within 48 hours of notification. Furthermore, focused investigations and responses are expected to be carried out within 7 days of case detection (Fig 1). These measures are essential for effective national malaria surveillance programs, as they enable the early detection of potential outbreaks and ensure the efficient deployment of control measures.

Achieving timely notification, and surveillance in endemic countries like Nepal comes with significant challenges. This is particularly true for remote areas that are difficult to access and lack connectivity, among other contextual factors. While improving infrastructure is vital for enhancing the speed of case reporting, there is a need to gather information on several aspects. This includes the duration between the onset of symptoms and diagnosis, the time taken for diagnosis and notification provided to the surveillance team, and the promptness of treatment received. Delays in patient, physician, and laboratory testing during outbreak concerns must be minimized in order to accomplish prompt detection and notification [7]. It is also important to examine whether these time intervals vary by any factors such as type of health facility, province, lab test, case classification etc. This information can then be used to improve the effectiveness and efficiency of the malaria surveillance system, leading to more timely interventions and ultimately supporting the goal of malaria elimination in the country. This study will help the country to gain a better understanding of the timeliness of malaria case notification and identify any potential factors influencing the notification process. Hence, this study was performed to address these knowledge gaps with the objective:

1. To conduct a timeliness analysis of malaria reporting, examining the duration between
  ❖ Symptom onset to diagnosis
  ❖ Diagnosis to notification
  ❖ Diagnosis to treatment
  ❖ Diagnosis to case investigation
2. To identify factors associated with the delayed notification of malaria in Nepal.

## Methods

### Study design

This study is a retrospective analysis of the data available from surveillance system of Epidemiology and Disease Control Division, under Ministry of Health and Population, Government of Nepal. The data is extracted from records maintained by EDCD.

### Study period

The study encompasses a timeframe of five years, spanning from 2018 to 2022. It was carried out during the months of June through September 2023, with the data being retrieved on June 1st, 2023.

### Study area

Nepal is a landlocked country in South Asia. It borders the Tibet, Autonomous Region of China to the north, and India in the south, east, and west. Nepal comprises three distinct geographic regions. Malaria vectors are notably found in the flat land and hilly regions, and a majority of the country’s population resides in these 2 regions. Not only this, from the Himalayan region i.e., above 4000 meters from sea level, indigenous cases are being reported, but only from some pocket areas like river valleys and the evidence of vector presence has been noted. The river flows from North (from the Himalayas) to South (towards hilly and finally to the Terai region), hence making a lot of river valleys in the Himalayan region as well. These river valleys have a favorable environment for vector growth.

### Sample size

The analysis did not use a pre-determined sample size; instead, all recorded malaria positive cases during the study period were included. Any missing or undocumented information in variables in the extracted data were excluded from further analysis, leading to different sample sizes for different analyses.

### Sample collection

#### Data source

The Epidemiology and Disease Control Division (EDCD) of the Ministry of Health and Population (MoHP) in Nepal has developed a surveillance system for malaria to ensure the complete recording and reporting of all positive malaria cases across Nepal. Malaria Disease Information System (MDIS) data of (2018–2022) is used for this analysis and is obtained from the Neglected Tropical Disease and Vector Borne Disease Control Section (NTDs/VBDs) EDCD database, after receiving the permission of the concerned department. EDCD routinely performs the data cleaning and management of the received information from all the platforms and saves it on the EDCD database.

#### Data collection

The variables in this dataset were originally collected by surveillance officers or malaria focal personnel or health workers at the provincial, district, or municipal levels using case investigation form developed for routine surveillance activities. The data collection process involved personal visits by these healthcare workers, either at hospitals for admitted patients or at the homes of discharged patients. Notably, all personal identifiers of both patients and healthcare workers were removed by EDCD to ensure confidentiality before making the data available for this analysis.

### Ethical consideration

This study was conducted utilizing pre-existing data collected by the National Malaria Elimination Program, Epidemiology and Disease Control Division as part of routine surveillance activities from 2018 to 2022. The data were anonymized and provided to us by the EDCD, maintaining the confidentiality of the individuals involved. Given that our study exclusively relied on secondary data collected for public health surveillance purposes and did not involve any direct interaction with human subjects, we did not seek ethical approval for this specific analysis.

The study involves authors affiliated with the National Malaria Elimination Program and have access to the data before analysis, however, the data was anonymized before being provided to the research team. Furthermore, the access granted to our research team was strictly for the purpose of aggregate analysis and did not involve any access to individual-level data that could potentially identify participants.

### Data management and data analysis

The statistical analysis for this study was conducted using R Studio 2023.06.1 Build 524. Descriptive analysis was performed to present the distribution of basic characteristics of malaria-positive cases and the distribution of time intervals. Summary statistics, including mean, median etc. are used to describe the central tendency and variability of the intervals. Bivariate analysis using cross tabulations was performed to identify factors associated with the timeliness of reporting. Logistic Regression analysis was conducted to assess the extent of association between the identified factors and delayed notification.

## Results

### Demographic characteristics

Table 1 shows the demographic characteristics of the malaria positive cases from 2018 to 2022. There was a total of 3029 positive cases in the data set, of which majority were (2419, 79.9%) male. The Male: Female ratio was 3.96:1. Among 3023 malaria positive cases, nearly two-third (65%) of the positive cases were between the age of 20 to 49 years. Children less than 10years of age were 5.6% and adults ≥ 60 years were 4%. Over one-third (36.7%) of the malaria-positive cases were repatriate workers and nearly one-fifth (16.9%) worked as laborers within the country. Approximately 15% were farmers, and over one-tenth (12.5%) of the malaria positives were students. Nearly half of the positive cases (43.3%) between 2018 and 2022 were from Sudurpaschim province. Over a quarter (25.6%) of the malaria positives were from Lumbini province. The 3rd highest burden province was the Karnali province, with 14.9% of the total positives during the mentioned 5 years. Bagmati Province, Gandaki, and Koshi province accounted for less than 5% of the total positive cases. Madhesh province reported 6.9% of the total positive cases.

**Table 1:** The demographic characteristics of the malaria positive cases, 2018–2022, Nepal.

Characteristics	N	Sub-category	n	%
**Sex**	3029	Male	2419	79.9
**Age group (Years) **	3023			
		< 10	169	5.6
		10–19	572	18.9
		20–29	991	32.8
		30–39	610	20.2
		40–49	380	12.6
		50–59	178	5.9
		60–69	83	2.7
		>70	40	1.3
**Occupation **	2962			
		Repatriate worker^a^	1087	36.7
		Labor	502	16.9
		Farmer	442	14.9
		Student	370	12.5
		Housewife	171	5.8
		Security Personnel	121	4.1
		Other	269	9.1
**Provinces**	3029			
		Sudurpaschim	1311	43.3
		Lumbini	775	25.6
		Karnali	451	14.9
		Madhesh	209	6.9
		Bagmati	119	3.9
		Gandaki	105	3.5
		Koshi	59	1.9

^a^ = Repatriate worker are those workers returning from abroad who majorly work as laborers, work in agriculture farm, water factories etc.

### Description of malaria positive cases

Table 2 presented the case detection methods, the laboratory tools utilized, and the species diagnosed for malaria cases in the past. The majority of malaria-positive cases (88.3%) were detected through passive case detection, while slightly more than one-tenth (11.7%) were identified through active case detection. The prevailing diagnostic approach involved the combined use of microscopy and Rapid Diagnostic Kit (RDT), accounting for 66.8% of the cases. Approximately a quarter (22.7%) of the positives were diagnosed exclusively with RDT, and just over one-tenth (10.5%) of the malaria-positive cases relied solely on microscopy for diagnosis.

**Table 2:** Description of malaria positive cases, 2018–2022, Nepal (N = 3029).

Variables	Sub-category	N	n	%
**Case Detection Method**		3029		
	Passive Case Detection		2674	88.3
	Active Case Detection		355	11.7
**Laboratory tools**		3029		
	Microscopy and RDT		2023	66.8
	RDT only		688	22.7
	Microscopy Only		318	10.5
**Species Type**		3024		
	P. Vivax		2530	83.7
	P. Falciparum		275	9.1
	P. Mix		212	7
	P. Ovale		7	0.2
**Case Classification **		2999		
	Imported ^a^		2221	74.1
	Indigenous ^b^		778	25.9
**Follow up**		2749		
	Yes		1576	57.3

^a^ Imported case: An imported case is one that is due to mosquito-borne transmission and is acquired in another country. National Malaria Program has standardized the initial classification of ‘imported’ malaria as a confirmed case of malaria detected within 1 month of return from an endemic area outside Nepal.

^b^ Indigenous case: Any case contracted within the country, with no evidence of a direct link to an imported case.

The most prevalent malaria species was Plasmodium vivax (83.7%), followed by Plasmodium falciparum (9.15%) and Plasmodium mix (7%). There were also a few instances (0.2%) where Plasmodium ovale was diagnosed. Nearly three-fourth of the cases were imported and more than half of the positive cases (57.3%) received at least one follow-up after the initiation of treatment.

**Fig 2:**
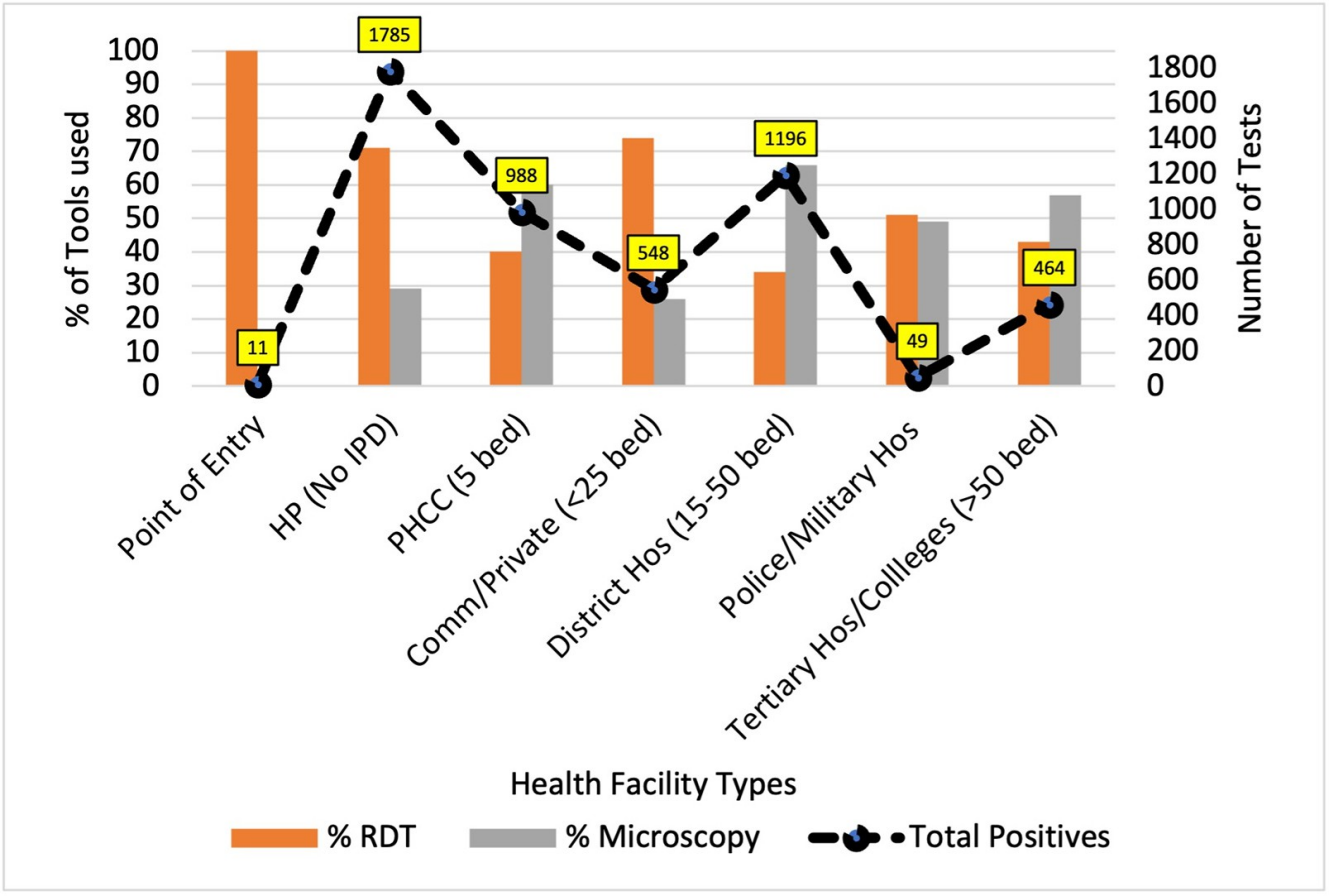
Health facility types and distribution of malaria positives by Tool, 2018–2022, Nepal.

Fig 2 provides an overview of the methods of diagnosing malaria cases by different facilities. It depicted that the Point of Entry (POE) exclusively used Rapid Diagnostic Tests kits (RDT) and successfully identified 11 cases among individuals returning from India. Health posts, primarily utilized RDT to diagnose approximately 70% of the nearly 1,800 cases detected over the past 5 years. Primary Health Care Centers (PHCC), equipped with medical doctors and offering inpatient care, diagnosed almost 1,000 cases, with approximately 60% of them relying on microscopy for diagnosis. Community and small private hospitals with fewer than 25 beds diagnosed over 500 cases, with a heavy dependence on RDT (around 75%). District hospitals diagnosed nearly 1,200 cases, and microscopy played a significant role in approximately 70% of this diagnosis. In contrast, the military hospital had fewer than 50 cases diagnosed, while tertiary centers managed to identify over 450 cases, with microscopy contributing to nearly 60% of the diagnosis.

### Duration and measurement of timeliness

Table 3 provides the information on time duration and their timeliness status. The median duration from the onset of symptoms to diagnosis was 5 days with the first quartile (Q1) of 3 days and the third quartile (Q3) of 8 days. Diagnosis to notification had a median duration of zero days, indicating that notifications were sent on the same day as diagnosis. Nearly 85% of these notifications were dispatched within 24 hours. Diagnosis to treatment also had a median duration of zero days with about 95.8% timeliness of cases receiving treatment within 24 hours of diagnosis. Regarding the time from diagnosis to case-based investigation, the median duration was 2 days, with Q1 at 1 day and Q3 at 2 days. 90% of these case-based investigations were adhered to the surveillance guideline, i.e., within 3 days of diagnosis.

**Table 3:** Duration and measurements of timeliness of malaria related information, 2018–2022, Nepal.

Stages	N ^a^	Min (days)	Q1 (days)	Median (days)	Q3 (days)	Max (days)	Timeliness (n ^b^)	Timeliness (%)
Symptoms onset to Diagnosis	2778	0	3	5	8	30	-	-
Diagnosis to Notification	2547	0	0	0	1	30	2161	84.8
Diagnosis to Treatment	2413	0	0	0	0	8	2312	95.8
Diagnosis to Case based investigation	2893	0	1	2	2	30	2611	90.2

^a^ Malaria positive cases with specific variable information in the extracted dataset (2018–2022), ^b^ Malaria-positive cases where timeliness (day 1 for notification and treatment, day 3 for case-based investigation) is followed

Table 4 displays the yearly distribution of the time taken from diagnosis to notification, treatment and case-based investigation. The median notification time has remained consistent (zero days) over the five-year period indicating that notifications were sent on the same day of diagnosis. Over the years, there has been a reduction in the maximum notification time dropping from 28 days in 2018 to 16 days in 2022. The median days from diagnosis to treatment remain the same day as diagnosis and there has been no improvement in the maximum days over the years. There has been fluctuation in the maximum duration for initiating case-based investigation over the years. And, increase in median days in recent years compared to 2018.

**Table 4:** Yearly distribution of time intervals (days) from malaria diagnosis to notification, treatment, and case-based investigation, 2018–2022, Nepal.

Year	N^a^	n^b^	n^c^	n^d^	Minimum			Q1			Median			Q3			Maximum		
					N^e^	T^f^	I^g^	N^e^	T^f^	I^g^	N^e^	T^f^	I^g^	N^e^	T^f^	I^g^	N^e^	T^f^	I^g^
2018	1019	640	523	927	0	0	0	0	0	0	0	0	1	1	0	2	28	8	27
2019	697	648	634	684	0	0	0	0	0	1	0	0	2	1	0	2	30	6	30
2020	429	396	410	408	0	0	0	0	0	1	0	0	2	1	0	2	29	4	19
2021	375	369	357	373	0	0	0	0	0	1	0	0	2	1	0	2	22	7	27
2022	509	494	489	501	0	0	0	0	0	1	0	0	2	1	0	2	16	8	24

^a^ Total malaria positive in respective year,

^b^ Malaria cases with documented diagnosis and notification date,

^c^ Malaria cases with documented diagnosis and treatment dates,

^d^ Malaria cases with documented diagnosis and case-based investigation dates,

^e^ Time interval (days) taken for notification from diagnosis,

^f^ Time interval (days) taken for treatment from diagnosis,

^g^ Time interval (days) taken for case-based investigation from diagnosis

### Malaria timely notification by province

**Fig 3:**
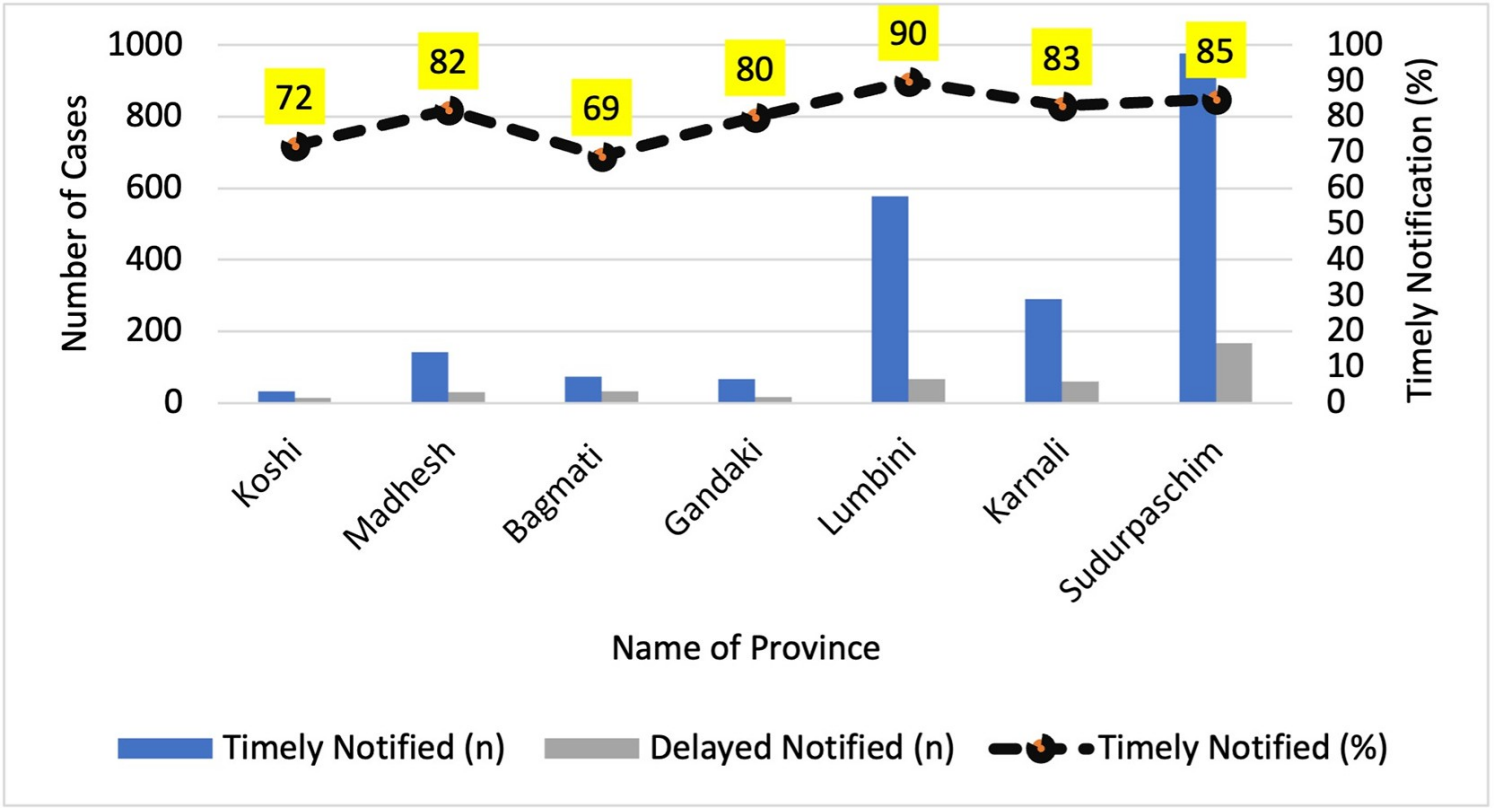
Malaria timely notification by province, 2018–2022, Nepal.

Fig 3 displays the timely notification of malaria cases by province. High-burden provinces, such as Lumbini province, Sudurpaschim province, and Karnali province have shown timely notification rates ranging from 83% to 90%. On the other hand, Bagmati province, a low burden province exhibits the least timely reporting.

### Univariate analysis

Table 5 presents the results of univariate analysis examining the relationship between various independent variables with delayed notification of malaria. Age showed a significant relation to delayed notification. Regarding occupation, repatriate workers, and students displayed significant association with delayed notification. All provinces, except Koshi and Gandaki province showed a significant association with delayed notification.

**Table 5:** Univariate analysis of independent variables with delayed notification.

Variables	Sub- Cat	Timely Notification (n %)	Delayed Notification (n%)	Crude OR	95% CI	P Value
**Gender**	Female	394 (82.4)	84 (17.6)	Reference		
	Male	1776 (85.4)	302 (14.6)	0.8	0.61, 1.05	0.10
**Age Group**	≤ 15	264 (89.5)	31 (10.5)	0.72	0.48, 1.08	0.12
	16–30	1088 (86.1)	176(13.9)	Reference		
	31–45	547 (82.1)	119 (17.9)	**1.34**	**1.04, 1.73**	**0.02**
	46–60	203 (82.2)	44 (17.8)	1.33	0.93, 1.92	0.11
	≥ 61	56 (77.8)	16 (22.2)	1.76	0.99, 3.14	0.05
**Occupation**	Repatriate Workers	891 (89)	110 (11)	0.55	0.39, 0.77	**<0.05**
	Labors	308 (82.4)	66 (17.6)	0.96	0.66, 1.41	0.87
	Farmers	294 (81.9)	65 (18.1)	Reference		
	Other	156 (76.1)	49 (23.9)	1.42	0.93, 2.15	0.10
**Province**	S-Paschim	977 (85.4)	167 (14.6)	**0.38**	**0.24, 0.59**	**<0.05**
	Lumbini	577 (89.7)	66 (10.3)	**0.25**	**0.15, 0.41**	**<0.05**
	Karnali	291 (83.1)	59 (16.9)	**0.45**	**0.27, 0.74**	**0.05**
	Madhesh	142 (82.1)	31 (17.9)	**0.48**	**0.27, 0.86**	**<0.05**
	Bagmati	74 (69.2)	33 (30.8)	Reference		
	Gandaki	67 (79.8)	17 (20.2)	0.56	0.29, 1.11	0.10
	Koshi	33 (71.7)	13 (28.3)	0.88	0.41, 1.89	0.74
**Case Detection Method**	PCD	1899 (84.6)	346 (15.4)	1.19	0.84, 1.7	0.32
	ACD	262 (86.8)	40 (13.2)	Reference		
**Laboratory Tools**	Microscopy Only	193 (78.1)	54 (21.9)	**1.99**	**1.43, 2.79**	**<0.05**
	RDT only	417 (78.4)	115 (21.6)	**1.97**	**1.53, 2.53**	**<0.05**
	Both	1551 (87.7)	217 (12.3)	Reference		
**Species Type**	P. Vivax	1750 (84.3)	326 (15.7)	1.1	0.76, 1.58	0.59
	P. Falciparum	225 (85.6)	38 (14.4)	Reference		
	P. Mix	182 (89.2)	22 (10.8)	0.71	0.40, 1.25	0.24
**Case Class**	Imported	1629 (85.1)	285 (14.9)	Reference		
	Indigenous	511 (84)	97 (16)	1.08	0.84, 1.39	0.52
**Health Facility Type**	Police/Military Hospital	21 (65.6)	11 (34.4)	**3.26**	**1.54, 6.90**	**0.05**
	District Hospital/DPHO	344 (81.7)	77 (18.3)	**1.39**	**1.03, 1.88**	**0.02**
	Health Post	972 (86.2)	156 (13.8)	Reference		
	PHCC	339 (90.9)	34 (9.1)	**0.62**	**0.42, 0.92**	**0.01**
	Community/Private hospital	300 (81.7)	67 (18.3)	**1.39**	**1.01, 1.90**	**0.03**
	Tertiary Centers	183 (82.4)	39 (17.6)	1.32	0.90, 1.95	0.14

For the case detection method and laboratory tools concerning delayed notification, it was found that laboratory tools had a significant association with delayed notification, while the case detection method did not. All the types of health facilities came out to be significantly associated with the delayed notification except tertiary centers.

### Multivariate analysis: Factors associated with delayed notification

In the final multivariate logistic regression model, variables with a P-value ≤ 0.2 from the univariate analysis were considered. Table 6 only displays the variables that remained significant after adjusting for gender, age, and health facility types during multivariate analysis. Occupation was identified as significantly associated with delayed notification. Malaria-positive individuals with the occupation type "repatriate worker" had lower odds of 0.60 (95% CI 0.41 to 0.87), of experiencing delayed notification compared to those whose occupation was farmer.

**Table 6:** Multivariate analysis for factors associated with delayed notification, 2018–2022, Nepal.

Variables	Sub Cat	Adjusted OR	95% CI	P Value
**Occupation**	Repatriate Workers	**0.60**	**0.41, 0.87**	**<0.05**
	Labor	1.05	0.69, 1.60	0.79
	Farmer	Reference		
	Student	0.83	0.40, 1.71	0.61
	Housewife	1.46	0.83, 2.57	0.18
	Security Personnel	0.58	0.28, 1.23	0.15
	Other	1.21	0.76, 1.91	0.41
**Province**	S-Paschim	**0.48**	**0.29, 0.81**	**<0.05**
	Lumbini	**0.38**	**0.22, 0.67**	**<0.05**
	Karnali	0.65	0.36, 1.15	0.14
	Madhesh	0.63	0.34, 1.17	0.15
	Bagmati	Reference		
	Gandaki	0.64	0.31, 1.29	0.21
	Koshi	0.93	0.41, 2.10	0.87
**Laboratory Tools**	Both	Reference		
	RDT only	**2.04**	**1.55, 2.67**	**<0.05**
	Microscopy Only	**1.79**	**1.23, 2.59**	**<0.05**

Likewise, individuals diagnosed with malaria in Sudurpaschim and Lumbini provinces had significantly lower odds (0.48 and 0.38 respectively), of encountering delayed notification in comparison to those in Bagmati province. In other words, being diagnosed in S-Paschim and Lumbini province is identified as a protective factor against delayed notification. We found that relying on a single laboratory tool for malaria diagnosis (either RDT or Microscopy) is significantly associated to delayed notification. Individuals diagnosed only with RDT or microscopy had higher odds, 2.04 and 1.79 respectively, of experiencing delayed notification compared to those diagnosed with both RDT and microscopy.

## Discussion

This study showed that the median notification time has remained relatively stable, and over time there has been an improvement in the maximum notification time. The median time from diagnosis to notification was zero days, suggesting that notifications had been sent out on the same day as the diagnosis. Approximately 85 percent of these notifications had been sent out in a single day. In contrast to our results, a study done in Qatar showed that the median time lag of two days, found between the reporting of a suspected case and the receipt of the notification by the surveillance section at MoPH [8] which explained that Qatar’s reporting system is paper-based. A transition to electronic system resulted in early case notification in Ghana where timeliness of reporting increased from 45% to 61% [9]. Zero median time delay in notification in our study could be due to the advancement in the electronic reporting system via mobile phone (SMS based) reporting in case of malaria.

The median time lag between symptom onset and diagnosis was found to be 5 days, which was longer than the findings in Qatar which showed it to be 4 days [8]. This duration of symptom onset to diagnosis depends upon overall health seeking behavior which might have affected the duration in this study. Another study done in China-Myanmar border showed average duration from onset of symptoms to diagnosis to be 8.5 days which was due to frequent mobility and migration from open border between these two countries [10]. The median time in our study for diagnosing and case investigation of malaria positives was 2 days, which is within the 3-day window of the 1-3-7 surveillance approach. A study conducted along the China-Myanmar border suggests that it would be challenging to finish a case investigation in three days since it takes longer time to transport and process samples, which can delay the diagnosis and it is difficult to monitor the mobile population at the border [10].

In this study, a noteworthy lower odd of delayed notification among repatriate workers compared to individuals in different occupational categories was observed. This phenomenon could be attributed to the heightened awareness among healthcare workers regarding the prevalence of imported malaria, particularly in regions with a high malaria burden. Healthcare professionals may take the proactive step of reporting a positive case of malaria among returned workers, as our surveillance strategy clearly specifies.

The results also revealed that Sudurpaschim and Lumbini Provinces, characterized by a high malaria burden, exhibited lower odds of delayed notification. Conversely, Koshi, Bagmati, and Gandaki, provinces with a lower malaria burden, had a higher proportion of delayed notifications. However, in provinces with a low burden of malaria cases, notification delays may arise from a lack of awareness among healthcare personnel at primary healthcare centers and health posts. This difference can be attributed to the allocation of resources and focused efforts in areas with a high disease burden potentially leaving areas with fewer cases with less attention. In regions with lower disease burden, it is imperative to ensure that the healthcare workforce is adequately trained to suspect, diagnose, treat, and promptly report cases [11]. Furthermore, the allocation of resources for supervision, training, and monitoring should be tailored to address the specific needs of these provinces, enhancing their capacity to effectively manage and respond to malaria cases. This is supported by a finding in Korea where due to unfamiliarity of the disease both diagnosis and doctors report seem to be delayed [12].

This study revealed that diagnosis of malaria by using only RDT or microscopy have higher odds of delayed notification (2.04 and 1.79 respectively) compared to diagnosis using both RDT and Microscopy. This delay could be attributed to potential diagnosis dilemma when relying on a single test. RDTs offer rapid results and ease of use, they are qualitative in nature and may yield more false positives due to the continued presence of antigens and other factors [13, 14]. Similarly, the accuracy of microscopy depends upon the expertise of the microscopist and the quality of staining reagents [15]. The presence of a positive result from a single tool may have caused uncertainty among healthcare workers regarding the diagnosis, resulting in delayed notifications, as opposed to cases where both tests were positive. Higher number of notified cases have used both methods for diagnosis. Since each method has benefits and drawbacks of its own, an appropriate diagnosis should be made using a combination of both methods [16, 17].

### Limitation

- Retrospective nature and use of secondary data in the study could be the reason for bias since there could have been human error in the recording and reporting of the data.
- While this dataset incorporates both active and passive reporting, it’s worth noting that not all cases may have been identified or reported, especially those that are asymptomatic.
- Due to the retrospective nature of the study, there are incomplete records, data entry errors, and missing information that may have impacted the overall quality of the data, thereby limiting the scope of our analysis.
- The data was gathered continuously over time, which could potentially introduce temporal bias into our findings.
- Due to regional differences in reporting practices and access to health care, the data reported may exhibit geographic bias, making it difficult to generalize our conclusions to the broader population. Our ability to control for confounding variables in the data set was limited.

## Conclusion

There were no delays identified in the median notification, treatment, and case investigation times. It showed improvement in the timeliness in malaria reporting over the study period. Provinces with higher malaria burden and repatriate workers showed lower odds of delayed notification compared to other provinces and occupations. Cases diagnosed using a single laboratory tool (either malaria RDT or microscopy) experienced delays in notification compared to cases diagnosed using both tools.

### Recommendations

- Special emphasis should be placed on educating repatriate workers, a vulnerable population group and the provision of chemoprophylaxis could prove beneficial.
- Adjusting strategies and resources for provinces based on their respective malaria profiles is essential.
- Enhanced lab facilities with unified procedures for diagnosis is essential to optimize notification timeliness.
- Strengthening the healthcare system’s capacity to effectively track and follow up with patients, especially for ensuring radical cure, is imperative.
